# Osteolytic lesions (brown tumors) of primary hyperparathyroidism misdiagnosed as multifocal giant cell tumor of the distal ulna and radius: a case report

**DOI:** 10.1186/s13256-018-1723-y

**Published:** 2018-06-25

**Authors:** A. Panagopoulos, I. Tatani, H. P. Kourea, Z. T. Kokkalis, K. Panagopoulos, P. Megas

**Affiliations:** 1 Orthopaedic Department, Patras University Hospital, Papanikolaou 1, 26504 Rio-Patras, Greece; 2 Department of General Surgery, Patras University Hospital, Rio-Patras, Greece; 3 Department of Pathology, Patras University Hospital, Rio-Patras, Greece

**Keywords:** Brown tumor, Primary hyperparathyroidism, Giant cell tumor, Parathyroid carcinoma

## Abstract

**Background:**

Brown tumors represent a rare clinical manifestation reported in approximately 3% of patients with primary hyperparathyroidism and correspond to radiologically osteolytic lesions with well-defined borders in different parts of the skeleton.

**Case presentation:**

We report the case of a 53-year-old white man who presented to our hospital with osteolytic lesions of his distal ulna and radius, causing pain and swelling of 2-month duration. A subsequent biopsy revealed histological features consistent with giant cell tumor and a complete resection of his distal ulna was followed, along with curettage and cementoplasty of the distal radial metaphysis. Two weeks later, he was re-admitted with diffuse musculoskeletal soreness, anorexia, constipation, nausea, and localized abdominal pain and multiple osteolytic lesions on plain radiographs. A histopathological examination of the ulna and radius specimens showed similar findings and, given the multifocality, brown tumors related to primary or secondary hyperparathyroidism was included in the differential diagnosis. A laboratory examination showed high total serum calcium (14.5 mg/dl) and low serum phosphorus and 25-hydroxyvitamin D levels. Primary hyperparathyroidism was suspected and confirmed by the elevated parathyroid hormone levels of 1453 pg/mL. At radiological work-up, using computed tomography, ultrasonography, and parathyroid subtraction technetium-99m sestamibi scintigraphy, a 4.5 × 2.5 × 3.2 cm mass emanating from the right lobe of his thyroid gland was detected, displaying extensive uptake in the right lower parathyroid gland. After appropriate medical support including hyperhydration and high doses of diuretics and diphosphonates, his laboratory profile normalized and he underwent total thyroidectomy with removal of the parathyroid glands. Our patient is now recovering 12 months after surgery, with normal values of serum parathyroid hormone and calcium levels. The lytic bone lesions have almost disappeared and no other additional orthopedic intervention was necessary.

**Conclusions:**

The present case report emphasizes the need of inclusion of brown tumors in the differential diagnosis of multifocal osteolytic bone lesions, in order to avoid harmful surgical interventions. Laboratory testing of serum phosphate, calcium levels, and parathyroid hormone levels should always be included in the routine survey of patients with multifocal osteolytic lesions.

## Background

Brown tumor (BT) represents a pathologic expression of “osteitis fibrosa cystica” encountered in patients with uncontrolled hyperparathyroidism. This tumor-like lesion represents the terminal stage of the bone remodeling process in prolonged hyperparathyroidism and has an overall incidence of 2–3% [1, 2]. BTs can be located in any part of the skeleton, but most frequently they are found in the jaws, ribs, clavicles, extremities, and pelvic girdle; they may be invasive in some cases, but lack malignant potential [3, 4]. Clinical manifestations include swelling, pathological fracture, and diffuse skeletal pain; in cases involving multiple bones these lesions can occasionally be mistaken for metastatic disease [2, 5–11]. The diagnosis of BT is based on medical history, clinical examination, laboratory results, and radiological imaging; it requires a high index of suspicion. There are many similarities in the radiological and histological features of BTs and giant cell tumors (GCTs), but these lesions very rarely coexist [12]. Surgical biopsy is the gold standard for definitive diagnosis, but radiological findings and biochemical testing, including serum calcium, phosphorous, and parathyroid hormone (PTH) levels are also essential diagnostic tools [1, 2, 13]. Treatment of these tumors consists mainly of partial or complete resection of the parathyroid glands which is subsequently followed by spontaneous tumor regression [14, 15].

We present a case of a 53-year-old white man, initially misdiagnosed with GCTs of the distal ulna and radius, who subsequently underwent unnecessary distal ulna resection; he presented later with multiple BTs attributed to primary hyperparathyroidism (PHPT) from parathyroid gland carcinoma. This report emphasizes the importance of considering PHPT in the differential diagnosis of patients with multiple lytic bone lesions, thus avoiding unnecessary and harmful interventions.

## Case presentation

**Fig. 1:**
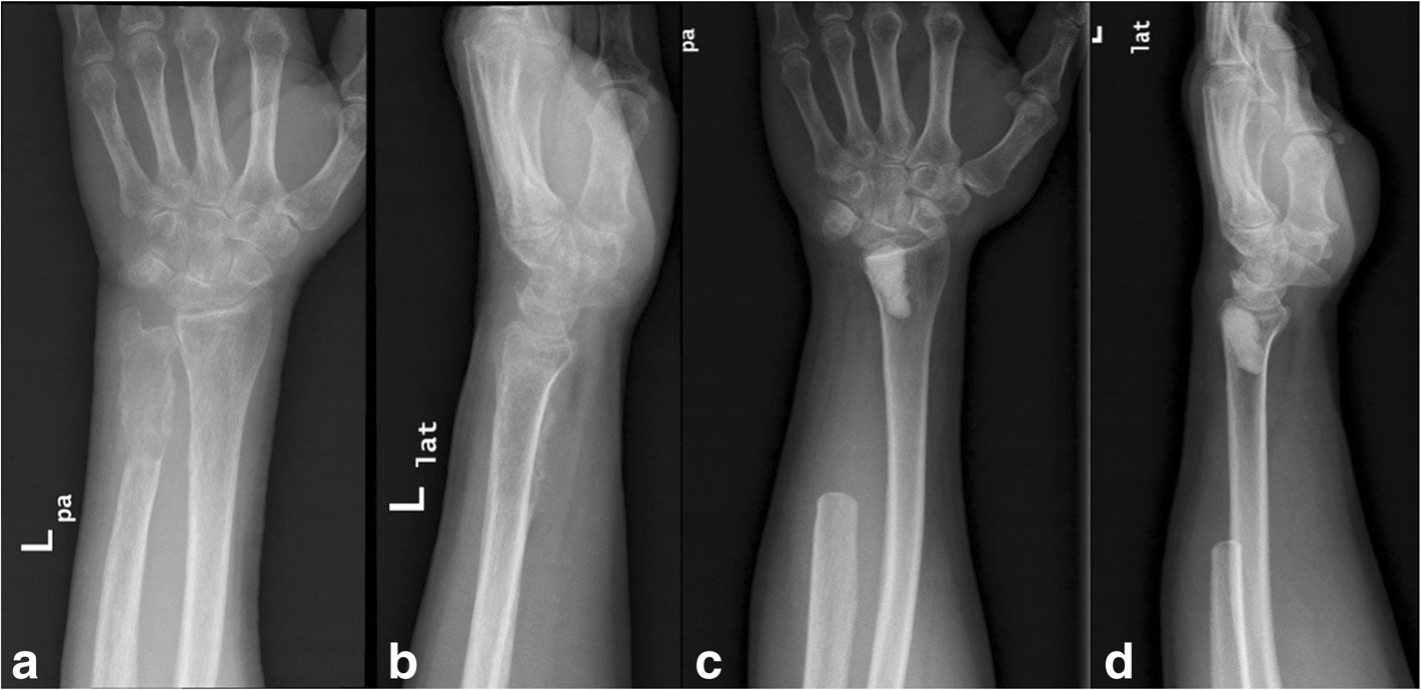
**a**, **b** Anteroposterior and lateral X-rays of patient’s left wrist showing an osteolytic lesion of the distal ulna with cortical expansion and disruption as well as an adjacent contained lesion in the lunate fossa of the distal radius. **c**, **d** Postoperative X-rays showing resection of the distal part of the ulna and curettage and cementoplasty of the distal radius. *lat* lateral, *pa* anteroposterior

A 53-year-old white man presented to our Department with a 2-month history of a painful and moderately swollen left wrist. His past medical history was unremarkable. Standard anteroposterior and lateral X-rays of his left wrist revealed two osteolytic lesions involving the distal ulna and the lunate fossa of the distal radius without any joint involvement (Fig. 1a, b). Subsequent biopsy of his left ulna under regional anesthesia produced brown spongy material, histologically characterized by the presence of large numbers of multinucleated giant cells and spindle cells in a dense collagenous background. These findings were histologically consistent with a diagnosis of GCT and correlation with the clinical and radiological findings was recommended by the pathologist. As he had no other skeletal manifestations, a complete resection of the distal ulna (9.5 cm length) followed, along with curettage and cementoplasty of the distal radial metaphysis, to support the articular surface (Fig. 1c, d). The resected distal ulna specimen and the curettings from the distal radius were submitted for histopathological evaluation; our patient was discharged 2 days later, with a forearm cast and instructions to attend the clinic in 2 weeks’ time for re-evaluation and removal of sutures.

**Fig. 2:**
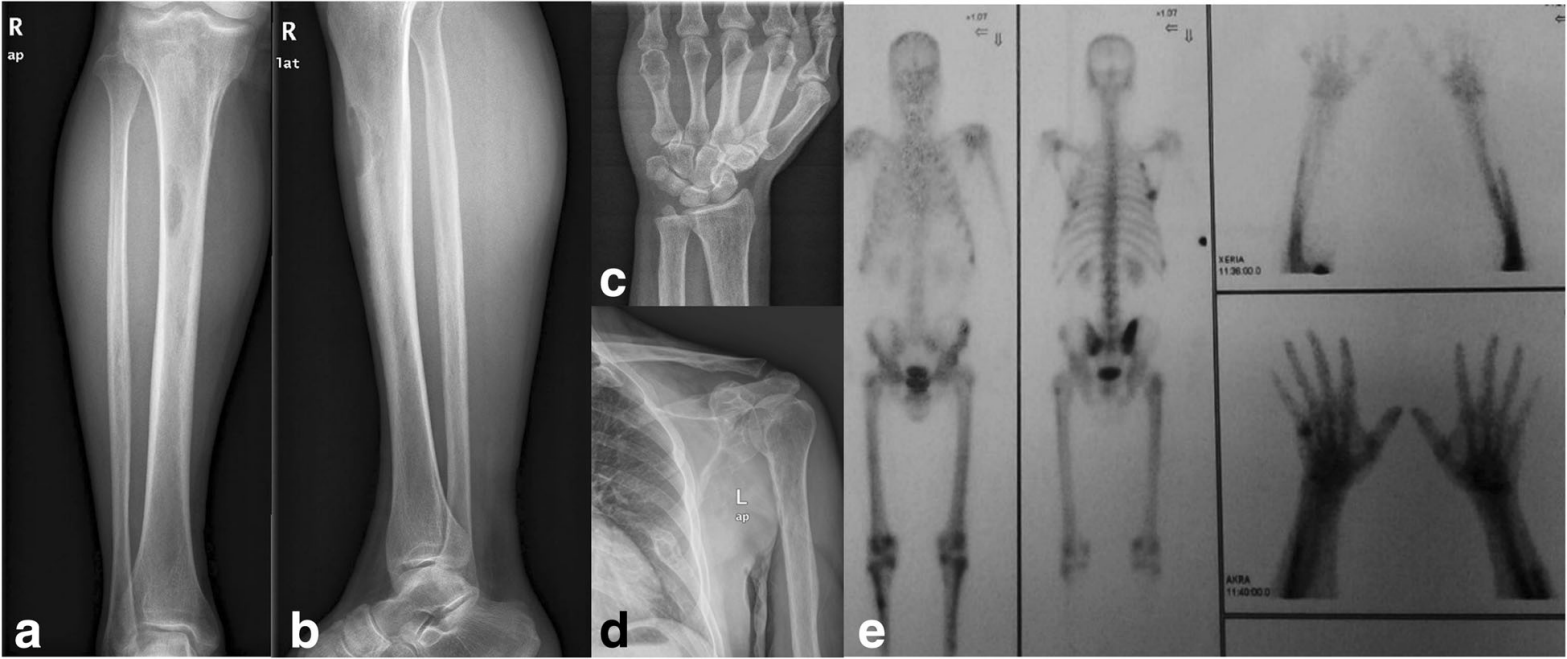
Radiological work-up after patient’s re-admission showing multiple osteolytic lesions at the right proximal tibia (**a**, **b**), head of fifth left metacarpal (**c**), and left shoulder (**d**). An isotope bone scan (**e**) showed multiple uptakes over the ribs bilaterally, the lower pole of both scapulae, multiple areas of the pelvis, the metacarpal bones of the right hand, and the right tibia.

Two weeks postoperatively, he was re-admitted to our orthopedic department with diffuse musculoskeletal soreness, anorexia, constipation, nausea, and localized abdominal pain. He also reported weight loss of approximately 5 kg. On palpation he had tenderness in the thoracic wall, the second and fifth metacarpals of his right hand, the left tibia, the pelvic ring, and the left shoulder girdle and humerus. Plain radiographs revealed multiple osteolytic lesions in his ribs, right hand, left tibia, and scapula (Fig. 2a–e).

**Fig. 3:**
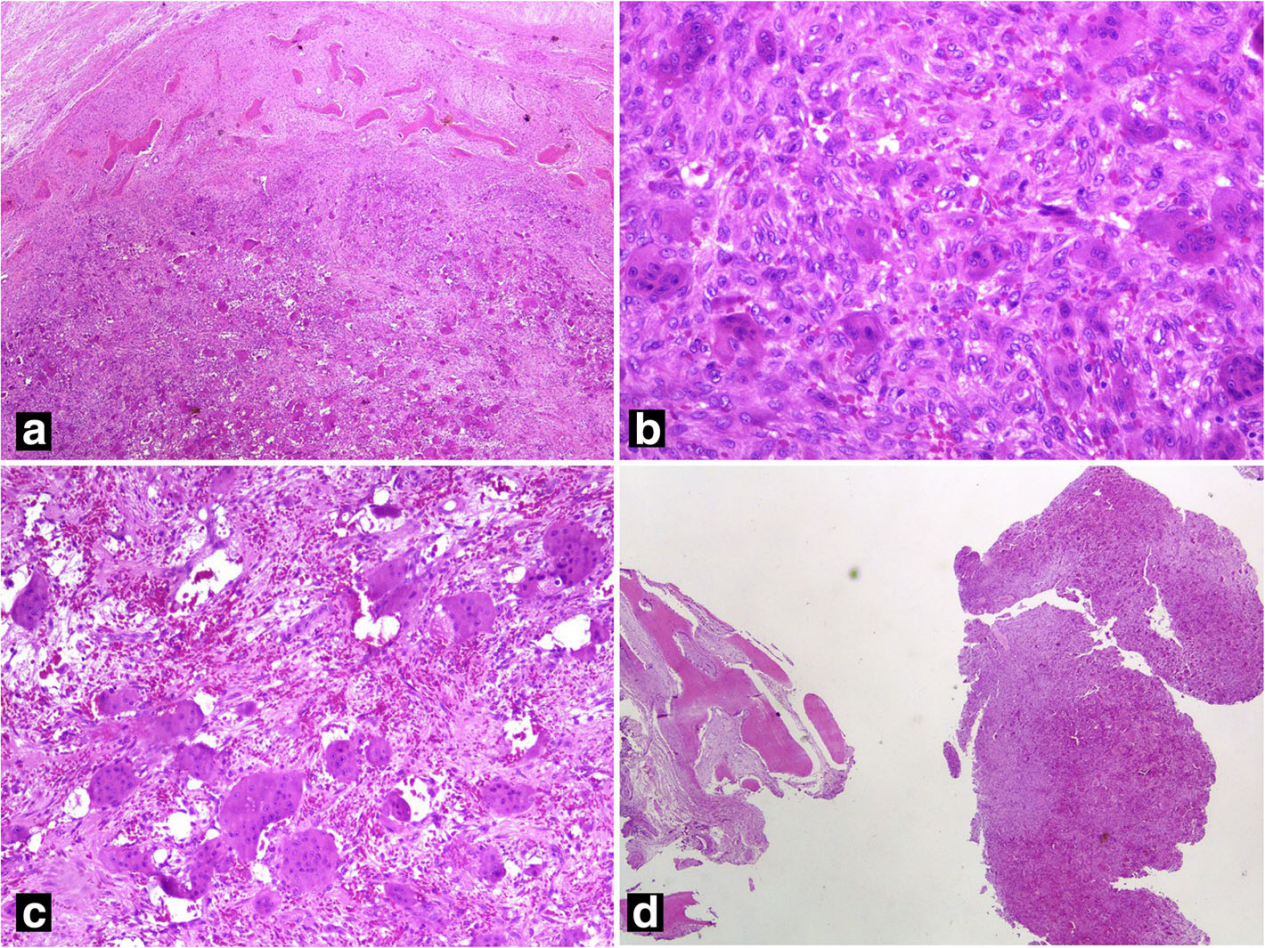
**a** In the resected ulna, the lesion disrupted the cortex leading to periosteal reaction with woven bone trabeculae. **b** Multinucleated, osteoclast-type giant cells were scattered among mononuclear spindle cells lacking atypia or sarcomatoid features. **c** Giant cell clusters with surrounding prominent foci of hemorrhage and **d** curettings of the radius showing similar morphologic findings

A histopathological examination of both the resected ulna (Fig. 3a–c) and the curettings of the radius (Fig. 3d) revealed similar findings: numerous, multinucleated, osteoclast-type giant cells were noted amid a mononuclear, spindle cell, histiocytoid component (Fig. 3b). Many of the giant cells were clustered in large nodular aggregates separated by fibrous septa containing fibroblast-like spindle cells. The spindle cell component showed no evidence of atypia or sarcomatoid features (Fig. 3c). There were prominent foci of hemorrhage with relatively restricted hemosiderin deposition (Fig. 3c). Mitoses were observed (up to five mitotic figures/ten high power fields) but no atypical mitoses or necrosis were seen. On the resection specimen of the ulna, the lesion focally disrupted the cortex producing periosteal reaction with woven bone trabeculae, extending in the surrounding adipose tissue and skeletal muscle (Fig. 3a). Based on the similar findings of both lesions and the rarity of multifocal GCT of bone the histopathology report included in the differential diagnosis a BT of hyperparathyroidism, either primary or in the setting of a paraneoplastic PTH-like protein production and suggested further patient evaluation.

**Fig. 4:**
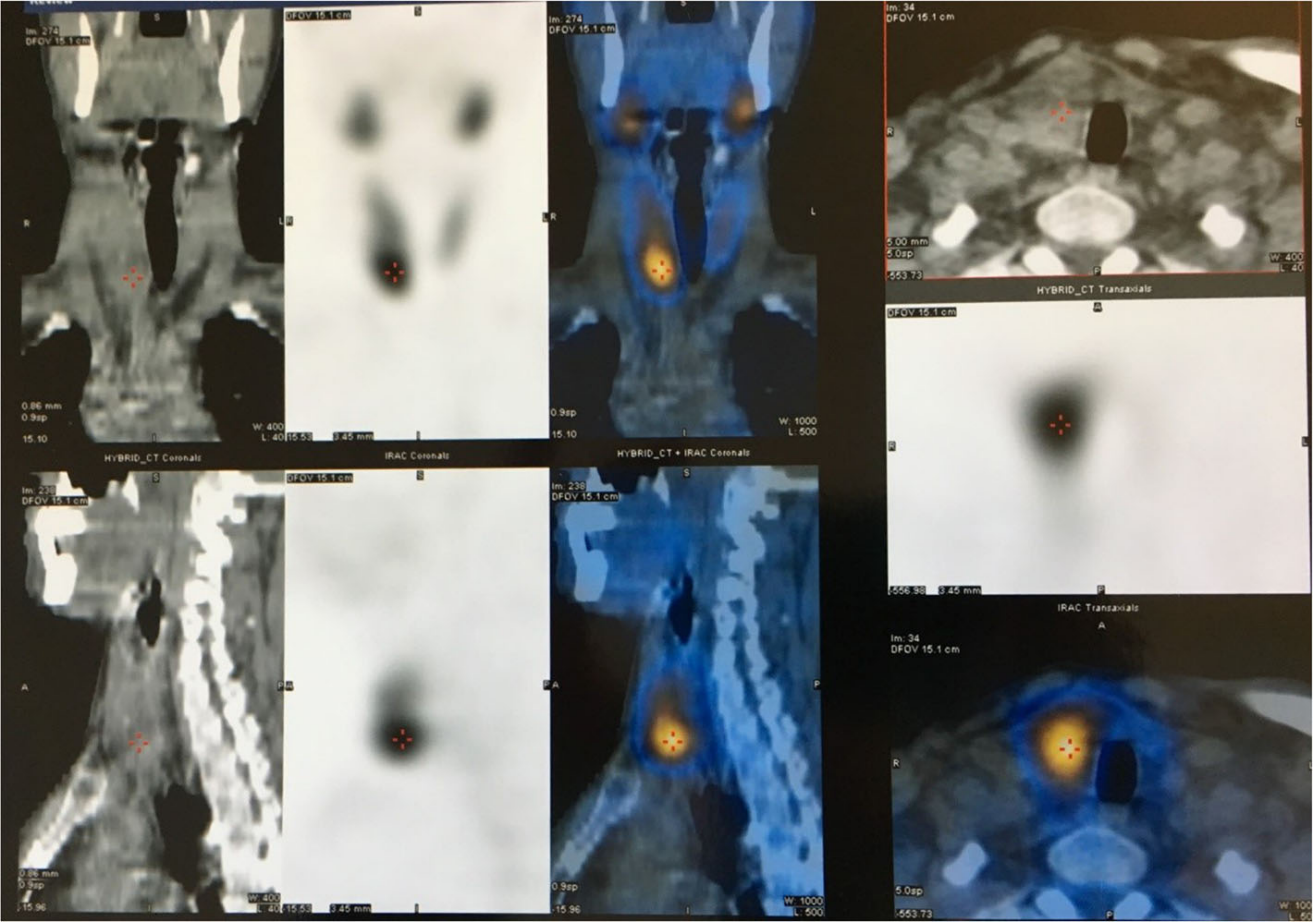
Computed tomography and ultrasonography of the neck revealed a 4.5 × 2.5 × 3.2 cm mass emanating from the right lobe of the thyroid gland. Parathyroid subtraction technetium-99m sestamibi (MIBI) scintigraphy showed extensive uptake in the right lower parathyroid gland

Our patient’s laboratory examination showed high total serum calcium (14.5 mg/dl, normal range 8.8–10.4), low serum phosphorus (2.3 mg/dl, normal range 2.5–4.5), and low 25-hydroxyvitamin D (9.74 ng/ml, normal range > 30). PHPT was suspected and confirmed by the elevated PTH levels of 1453 pg/mL (normal range 15–65). Serum potassium and sodium concentrations and thyroid hormone levels were in reference range as well as the main cancer indicators: cancer antigen (CA) 15-3, carcinoembryonic antigen (CEA), CA 125, and prostate-specific antigen (PSA). Serum protein electrophoresis was also normal. His human chorionic gonadotropin (hCG) was elevated (25.3 mUl/ml, reference level < 5). An isotope bone scan showed multiple sites of uptake over his ribs bilaterally, the lower pole of both scapulae, multiple foci in his pelvis, the metacarpal bones of his right hand, and his right tibia. At subsequent radiological work-up, both computed tomography (CT) and ultrasonography of his neck revealed a 4.5 × 2.5 × 3.2 cm mass emanating from the right lobe of his thyroid gland. Parathyroid subtraction technetium-99m (^99m^Tc) sestamibi (MIBI) scintigraphy showed extensive uptake in his right lower parathyroid gland (Fig. 4). Multiple endocrine neoplasia was excluded because of the normal MRI imaging of his pituitary gland.

Appropriate medical care was given to our patient including hyperhydration and high doses of diuretics and diphosphonates. After his health status improved and his serum calcium nearly normalized, a specialist surgeon was consulted for further surgical treatment. Surgery consisted of extensive resection: total thyroidectomy with removal of the parathyroid glands. A mass measuring 4.8 cm in greatest diameter, abutting the thyroid gland was documented at surgery. The mass was surrounded by a thick capsule, had a tan-brown, solid, and microcystic cut surface, and rubbery consistency. On histologic examination, the tumor comprised small cells with minimal to scanty cytoplasm and round nuclei, arranged in an organoid pattern, with frequent perivascular pseudorosettes. Thick fibrous septa emanating from the capsule were noted within the tumor. There was capsular invasion, with extension of neoplastic groups in the surrounding loose connective tissue adjacent to striated muscle, and foci of vascular invasion in the tumor capsule. The histologic findings were consistent with a parathyroid carcinoma. The neoplasm did not appear to invade the adjacent thyroid lobe and did not involve the margins of resection.

**Fig. 5:**
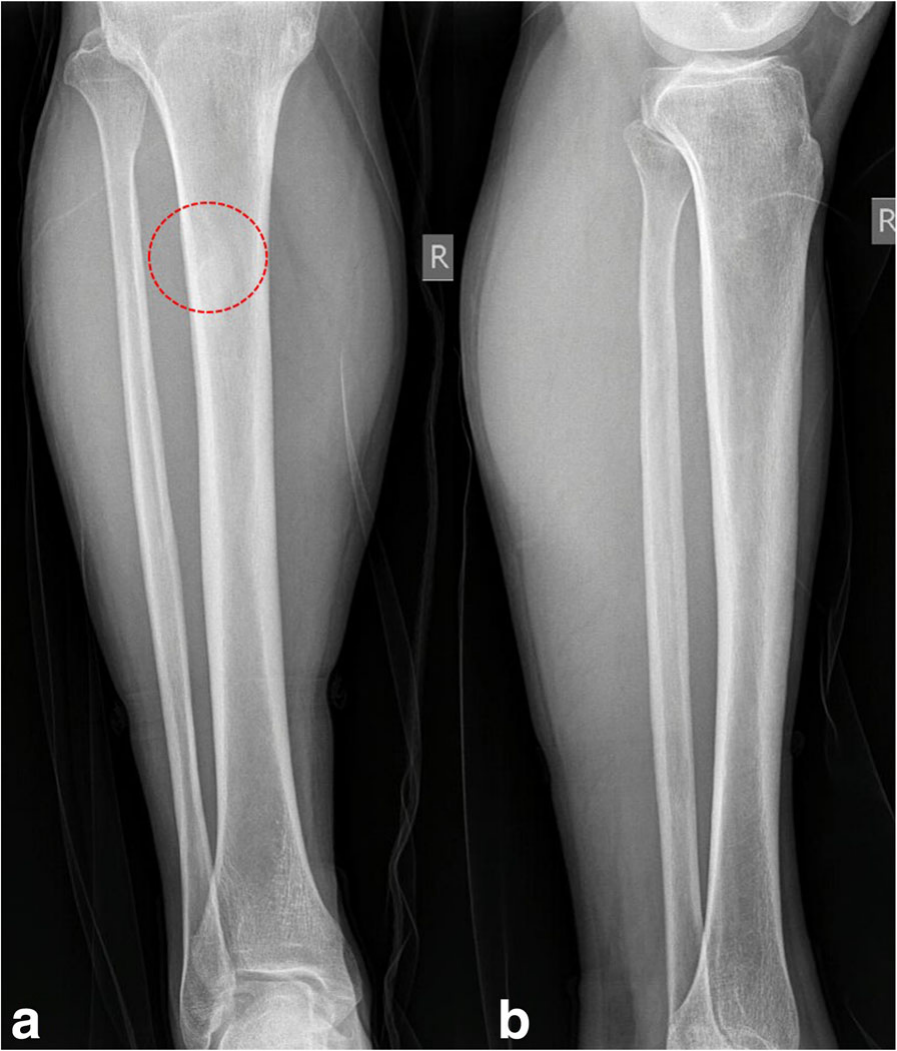
**a**, **b**Lateral radiographs of the right tibia (see also Fig. 1) at 1-year follow-up showing regression of the brown tumor (*red dotted area*)

Our patient experienced postoperatively persistent hypocalcemia requiring calcium and vitamin D replacement. His condition was characterized as “hungry bone syndrome.” He is now recovering 12 months after surgery, with a serum PTH level of 7.1 pg/mL and serum calcium level of 10.7 mg/dl and he is under calcium and vitamin D replacement therapy. The lytic bone lesions have almost disappeared (Fig. 5a–c) and no other additional orthopedic intervention is necessary. He is closely followed by general surgeons, oncologists, and endocrinologists.

## Discussion and conclusions

BTs represent a rare clinical manifestation of prolonged hyperparathyroidism (primary, secondary, or tertiary), reported in approximately 3% of patients with PHPT and 2% of those with secondary hyperparathyroidism [16–18]. On radiological examination they appear as osteolytic lesions with well-defined borders; their differential diagnosis includes primarily bone metastasis, amyloid cysts, chondroma, aneurysmal bone cyst, osteosarcoma, and GCT or myeloplax tumors [16, 18]. PHPT is the third most common endocrine disease after diabetes and thyroid disease with the highest incidence in postmenopausal women [13, 19]. The main causes of this condition are a solitary adenoma in 80–85% of patients, multiple adenomas in 5%, parathyroid hyperplasia in 10–15%, and carcinoma in less than 1–5% [20].

Nowadays, PHPT is mostly an asymptomatic mild disease, which is usually diagnosed by routine biochemical screening and not by the presence of typical clinical signs suggesting the disease [21]. Bone is a classic target organ in PHPT, and common skeletal changes involve bone reabsorption, cysts, and diffuse osteopenia. PHPT diagnosed in the context of osteitis fibrosa cystica or multiple BTs is an extremely rare condition nowadays, except in cases of severe untreated PHPT or in association with parathyroid carcinoma. Diagnostic dilemma can arise when the clinician faces multiple lytic bone lesions at different areas of the skeleton, as in the present case. Our patient had an initial biopsy result of GCT, which rarely presents multifocally, and underwent a distal ulna resection, which was unnecessary. A more serious underlying disease was responsible for his deterioration during the 2-weeks post-surgery. Metastatic bone lesions, multiple myeloma, or a metabolic bone disorder were considered in the differential diagnosis.

There are several reports in the literature of BTs mimicking bone metastases (2, 5–11). Despite the multiplicity of the lesions detected during his second admission, our patient had only mild skeletal pain and did not use narcotics for pain relief. On the other hand, the increased uptake on a whole-body bone scan and the negative serum protein electrophoresis rendered multiple myeloma less likely. His significantly elevated serum calcium and PTH levels were the clues that pointed our attention toward metabolic bone disease. Although hypercalcemia with high PTH levels and radiological evidence of a parathyroid gland tumor indicated BTs as a highly possible scenario, the correlation of clinical, laboratory, and histopathological findings established the diagnosis definitively. The increased secretion of PTH from the parathyroid carcinoma triggered high osteoclast activity, leading to the multiple osteolytic bone lesions seen in our patient.

Of interest, there are several studies describing PHPT and BTs mistaken for GCTs [22–24]. Pezzillo *et al.* [22] described two mistaken cases of BT, which were an aneurysmal bone cyst in the femur and an isolated GCT in the humerus; Jouan *et al*. [23] described a patient with typical manifestations of PHPT who underwent unnecessary amputation of the fifth ray of his right hand; and, finally, Vera *et al*. [24] reported the case of a patient who had surgical ablation of a costal mass on his third right rib, misdiagnosed at histology as a GCT. BTs and GCTs share similar clinical and radiological findings and differential diagnosis can be extremely difficult. On histological examination, they are composed of intensely vascular fibrotic stroma with multinucleated giant cells; their histological similarity is a challenge for the pathologist to arrive at a definite diagnosis based only on the assessment of bone specimens. Therefore, it is important to provide the pathologist with all the appropriate laboratory and clinical data.

There have also been reports describing the concomitant presence of a GCT with BTs of hyperparathyroidism [12, 25]. In such cases a GCT must be suspected when no regression of the bony lesion is observed after appropriate treatment of the hyperparathyroidism. Rossi *et al.* [12] presented the case of a 37-year-old white woman affected by a GCT of her proximal left tibia and concomitant asymptomatic PHPT due to a parathyroid adenoma. The presence of two concurrent diseases complicated the diagnosis and appropriate treatment; she was first treated for the adenoma, and 9 months later she underwent curettage of a tibial GCT and administration of denosumab for 12 months. Ouzaa *et al*. [25] described a honeycomb osteolytic lesion in the distal radius in a 66-year-old woman, diagnosed with PHPT. At 1-year follow-up she had worsening clinical symptoms and increased osteolyses on X-rays. A subsequent biopsy revealed the presence of a defined non-encapsulated tumor, with nodular proliferation of histiocytoid-type round cells, associated with multinucleated giant cells, consistent with the diagnosis of GCT; curettage and cementoplasty with iliac bone grafting led to a successful final outcome.

To the best of our knowledge, the present case is a rare example of multiple BTs associated with parathyroid carcinoma and PHPT. Only a few similar cases have been reported in the literature [26–28]. Parathyroid carcinoma is one of the most rarely reported malignancies with approximately 2 new cases per 10,000,000 persons per year [29]. Up to 90% of these tumors are hormonally functional, leading to excessive PTH secretion which overstimulates osteoclastic activity. Hyperparathyroidism caused by parathyroid carcinoma is usually severe, with high PTH and serum calcium levels and severe bone involvement. Vitamin D deficiency is common in patients with PHPT and may be associated with more aggressive disease, as was seen in our patient. According to epidemiological studies, vitamin D-deficient patients with PHPT have a higher level of PTH and markers of bone turnover, multiple lytic bone lesions, and higher incidence of fractures than patients who are deficient in vitamin D only [29, 30]. HCG, especially the hyperglycosylated isoform, is considered an important serum marker in parathyroid carcinomas; in patients with malignancy, increased urinary hCG or a rise in urinary hCG levels appear to signal a more aggressive phase of parathyroid cancer [30, 31]. Despite their high diagnostic utility, Tc sestamibi scintigraphy and ultrasonography cannot distinguish between benign and malignant parathyroid neoplasms. In our case, only the biopsy of the excised parathyroid specimen confirmed the diagnosis of parathyroid carcinoma.

The key treatment of BTs is surgical removal of the hyperfunctioning parathyroid gland. After addressing the parathyroid cause, BTs are expected to regress or to completely resolve. In our case, after normalization of serum PTH level, the bony lesions resolved and required no further orthopedic consultation. Surgical resection of BTs is generally not recommended, but orthopedic interventions should be considered in cases of pathological fractures or extensive cortical involvement. The risk of pathologic fracture should be estimated according to Mirels’ criteria [31]. In the present case, the osteolytic lesion of the right tibia had a borderline Mirels’ score of 8 but we decided not to proceed to prophylactic fixation, keeping our patient under close monitoring with instructions of non-weight bearing.

In conclusion, this case report emphasizes the need of including BTs of PHPT in the differential diagnosis of multifocal osteolytic bone lesions, in order to avoid unnecessary and harmful surgical interventions. A high index of suspicion is required for diagnosing BT at early stages; although osteolytic metastases and multiple myeloma should be considered first, laboratory testing of serum phosphate, calcium levels, and PTH levels should be included in the routine survey of patients with multifocal osteolytic lesions. Our mistake in accepting the diagnosis of multifocal GCT based on the suggestive findings of the biopsy report only led to an unnecessary surgery and put our patient’s life at great risk from hypercalcemia. A multidisciplinary approach with close communication between orthopedic surgeons, pathologists, and radiologists is crucial to arrive at the correct diagnosis.
